# Extracellular Vesicle-Encapsulated miR-183-5p from Rhynchophylline-Treated H9c2 Cells Protect against Methamphetamine-Induced Dependence in Mouse Brain by Targeting NRG1

**DOI:** 10.1155/2021/2136076

**Published:** 2021-08-26

**Authors:** Yuting Zhou, Shilin Xiao, Chan Li, Zhijie Chen, Chen Zhu, Qichun Zhou, Jinying Ou, Jing Li, Yifei Chen, Chaohua Luo, Zhixian Mo

**Affiliations:** ^1^School of Traditional Chinese Medicine, Southern Medical University, Guangzhou 510515, China; ^2^School of Life Science, Guangzhou University, Guangzhou 510006, China; ^3^Department of Pharmacy, The Second Affiliated Hospital of Guangzhou University of Chinese Medicine, Guangzhou 510000, China; ^4^Central Laboratory, Southern Medical University, Guangzhou 510515, China

## Abstract

Methamphetamine (Meth) is a highly addictive substance and the largest drug threat across the globe. There is evidence to indicate that Meth use has serious damage on central nervous system (CNS) and heart in several animal and human studies. However, the connection in the process of Meth addiction between these two systems has not been determined. Emerging data suggest that extracellular vesicles (EVs) carrying behavior-altering microRNA (miRNAs) play a crucial role in cell communication between CNS and peripheral system. Rhynchophylline (Rhy), an antiaddictive alkaloid, was used to protect the brain and heart from Meth-induced damage, which has caught our attention. Here, we used Meth-dependent conditioned place preference (CPP) animal model and cell model to verify the protective effect of Rhy-treated EVs. Further, small RNA sequencing analysis, qPCR, dual-luciferase reporter assay, and transfection test were used to identify the key EVs-encapsulated miRNAs, isolated from cultured H9c2 cells with different treatments, involved in the therapeutic effect and the underlying mechanisms of Rhy. The results demonstrate that Rhy-treated EVs exert protective effects against Meth dependence through the pathway of miR-183-5p-neuregulin-1 (NRG1). Our collective findings provide novel insights into the roles of EVs miRNAs in Meth addiction and support their potential application in the development of novel therapeutic approaches.

## 1. Introduction

Methamphetamine (Meth) abuse, a highly addictive synthetic derivative of amphetamine, is one of the most widely abused illegal drugs with over 17.2 million users worldwide and is a major contributor to public health problem and economic consequences [1, 2]. Chronic Meth use can result in a series of mental and physical symptoms including anxiety, insomnia, mood disturbances, cognitive impairments, paranoia, hallucinations, and delusion [3]. Furthermore, emerging researches showed Meth exposure has not only caused serious damage to the central nervous system (CNS) but also caused cardiotoxicity [4]. The animal studies indicated that Meth abuse could lead to increased heart rate, myocardial infarction, and cardiomyopathy via stimulating the sympathetic nervous system or autonomic nervous system [5]. The recent postmortem study also indicated that pathological cardiovascular damage was found in 68% of Meth abusers [6].

However, the underlying crosstalk between CNS and cardiotoxicity of Meth dependence is still unclear and the effect of currently available medications for Meth addiction is far from ideal. Traditional Chinese Medicine (TCM), which is a holistic medicine and emphasizes the integrity of body on the internal homeostasis, has historically been used to treat drug addiction up till today in clinical practice, particularly in Asia [7]. Rhynchophylline (Rhy), a major ingredient extracted from the traditional Chinese herb *Uncaria rhynchophylla* (Miq.) Miq. ex Havil., exerts strong inhibitory effects on drug addiction. Our previous studies demonstrated that Rhy can reverse the conditioned place preference (CPP) effect induced by Meth in rats and zebrafish [8, 9]. In addition to antiaddictive effect, Rhy is often used to treat cardiovascular diseases. The preclinical studies show that Rhy could prevent cardiac dysfunction and ameliorates myocardial ischemia/reperfusion injury [10, 11]. These findings highlight the potential for the development of Rhy as antiaddiction drugs, exploring the crosstalk between the CNS and heart during the process of Meth addiction. However, a major question is what connective mechanisms underlie the inhibitory effect on Meth dependence of Rhy acting between the brain and the heart?

Accumulating evidence shows that extracellular vesicles (EVs) play a vital role in connection between CNS and heart in the process of drug addiction [12–14]. EVs with a lipid bilayer membrane structure capable of crossing the blood-brain barrier can participate in multiple physiological and pathological processes [15, 16]. It is worth to note that EVs are reported to regulate neuronal development and regeneration, participate in cell communication in neurodegenerative diseases, and modulate synaptic functions in the CNS [17, 18]. Astrocyte-derived EVs are likely neuroprotective and contribute to neural regeneration and plasticity [19]. Furthermore, neuronal EVs act as potential biomarkers of neurological dysfunction in alcohol or tobacco users [20]. In addition, EVs play an important role in mediating cell-cell communication and contain a variety of microRNAs (miRNAs), proteins, and DNA molecules, which act locally or are stably transferred to recipient cells [9, 21]. Notably, miRNAs, packaged by EVs, are closely associated with CNS diseases. It can be exemplified with the study on EVs-encapsulated miR-29, which has been showed to regulate morphine-mediated neuronal dysfunction by targeting platelet-derived growth factor-B [22]. Moreover, our previous study showed the expressions of some miRNAs were changed in heart in Meth-induced rats, whereas these miRNAs can be reversed by Rhy treatment [23]. It is thus essential to study the roles and mechanisms of action of cardiomyocyte-derived EVs-containing miRNA in Meth dependence and Rhy treatments.

The current study was conducted to understand the biological characteristics and the protective effects of Rhy-treated H9c2-EVs. Moreover, we examined miRNAs profile in the EVs and the potential downstream pathway involved in Meth abuse and Rhy intervention between CNS and heart. These were intended to identify the novel therapeutic mechanisms to consider for future TCM to promote recovery among drug abusers.

## 2. Materials and Methods

### 2.1. Cell Culture

The cardiomyoblast cell line H9c2 and neuronal precursor cell line HT22 were obtained from the FuHeng Biology, Shanghai, China. Cells were cultured in DMEM medium containing 10% (v/v) fetal bovine serum (FBS, Gemini, Calabasas, California, USA) and 0.5% (v/v) penicillin/streptomycin (Gibco, Grand Island, NY, USA) and incubated at 37°C and 5% CO_2_. H9c2 cells were treated with PBS (Con group), Rhy (100 *μ*M) (No. RCY-000359, purity ≥ 98%, Jiangxi Baicaoyuan Biology Technology Co., Ltd., Nanchang, China), or Meth (250 *μ*M) (Meth hydrochloride, No. 1212-9802, National Narcotics Laboratory, Beijing, China) and cultured for 48 h in DMEM medium containing 10% EVs-depleted FBS. The EVs-depleted FBS was previously ultracentrifuged for 14 h at 120,000*g* to remove EVs in FBS.

### 2.2. Isolation, Identification, and Labeling of Extracellular Vesicles

EVs were purified from cell culture supernatant by differential centrifugation as previously described [24, 25]. In brief, the culture supernatants were cleared of cell debris and large vesicles by sequential centrifugation at 300*g* for 10 min, 1000*g* for 10 min, and 10,000*g* for 30 min, followed by filtration using 0.22 *μ*m syringe filters. Then, the cleared sample was spun at 100,000*g* for 70 min to pellet the EVs, and supernatant was collected. The collected EVs were washed twice with PBS. A transmission electron microscope (TEM, Hitachi H7650 TEM, Tokyo, Japan) was used to identify the form of the EVs. EVs pellets were suspended in PBS for negative staining. Twenty microliters of EVs was placed onto carbon-coated copper grids and allowed to absorb for 2 min. The copper grids were washed by distilled deionized water and then allowed to semidry for 20 min at 25°C before observation in TEM. Nanoparticle tracking analysis (NTA, Software ZetaView 8.04.02, Inning am Ammersee, Germany) was used to measure EVs diameter and particle number. Fluorescence labeling of EVs was performed according to the PKH26 kit (Sigma-Aldrich, St. Louis, MO, USA) instruction manual. The labeled EVs were washed at 100000*g* for 1 h, and the EVs pellet was diluted in PBS and used for the uptake experiment. The labeled EVs were incubated with HT22 cells for 12 h at 37°C and 5% CO_2_. After incubation, the culture medium was discarded and HT22 cells were washed 3 times with PBS. The cells were stained with DAPI, incubated for 10 min at 25°C, and imaged with an inverted fluorescence microscope (Olympus, Tokyo, Japan).

### 2.3. Animals

Kunming mice (male, 15–17 g, aged 23–26 days) were obtained from Guangdong Medical Laboratory Animal Center (license key: SCXY2018-0002). Mice were acclimatized in a controlled specific pathogen free (SPF) environment (20–22°C, 55–60% humidity, 12 h light/dark cycle, ad libitum access to food and water, license key: SYXK2016-0167). All procedures involving animals were approved by the Ethics Committee on the Use and Care of Animals, Southern Medical University, China.

### 2.4. CPP Model Experiments

The CPP apparatus was separated into two equally sized compartments using a mobile partition. One compartment was painted black with a smooth bottom while the other was painted white with a rough bottom. Mice could move freely between the two compartments when the mobile partition was removed. CPP consisted of three phases. In the preconditioning phase, after 3 days of adaptive feeding, 42 male mice (aged 33–36 days on “day 3”) were subjected to baseline determination (natural place preference) and randomly divided into 7 groups: Con (sterile 0.9% physiological saline), Meth (2 mg/kg), Rhy (60 mg/kg) + Meth, Con-EVs (100 *μ*g per each) + Meth, Rhy-EVs (100 *μ*g per each) + Meth, Rhy-EVs + Con, and Rhy + Con. In the conditioning phase (days 4–9), at 08:00 each day, mice received a subcutaneous injection of Meth hydrochloride (2 mg/kg, subcutaneous, s.c.) or sterile 0.9% physiological saline (10 mL/kg, s.c.) and were immediately placed in the white compartment for 45 min. After an 8 h interval, at 16:00 pm, mice from all groups received a sterile 0.9% physiological saline injection and were immediately placed in the black compartment for 45 min. From days 6 to 9, at 20:00 pm, mice from the Rhy + Meth and Rhy + Con groups received a daily intraperitoneal injection of Rhy solution (60 mg/kg, intraperitoneal, i.p.) whereas those from Con-EVs + Meth, Rhy-EVs + Meth, and Rhy-EVs + Control received a daily intranasal administration of EVs secreted by PBS-treated or Rhy-treated H9c2 cells (100 *μ*g per each, i.n.). In the postconditioning phase, at 24 h after the last injection of Meth (day 10), mice from all groups were freed into the CPP apparatus. The amount of time spent by each mouse in the white compartment within a 15 min time-period in the CPP apparatus was recorded as the postconditioning time. The Panlab Smart 3.0 (Harvard Bioscience, USA) was used to analysis the locomotor activity of each group. All mice were sacrificed for brain tissue collection.

### 2.5. Hematoxylin and Eosin Stain of Hippocampal Tissues

The hippocampal tissues of CPP model animals were fixed in 4% paraformaldehyde for 24 h, embedded in paraffin, and cut into 5 *μ*m thick sections for hematoxylin-eosin (H&E) staining. Tissue sections were observed under an optical microscope and photographed.

### 2.6. Small RNA Library Construction and Sequencing of EVs from H9C2 Cells

Total RNA samples were obtained from EVs by using Trizol (Invitrogen, USA). EVs were extracted from cell suspensions of the Con, Meth, Rhy + Meth, and Rhy groups by using Ribo™ Exosome Isolation Reagent (Guangzhou Ribo Biotechnology Co., Ltd., Guangzhou, China). The quantity and integrity of RNA yield were assessed by using the Qubit®2.0 (Life Technologies, USA) and Agilent 2200 TapeStation (Agilent Technologies, USA) separately. 1 *μ*g total RNA of each sample was used to prepare small RNA libraries by NEBNext® Multiplex Small RNA Library Prep Set for Illumina (NEB, USA) according to the manufacturer's instructions. The libraries were sequenced by HiSeq2500 (Illumina, USA) with single-end 50 bp. RNA sequencing measurement was conducted by Guangzhou Ribo Biotechnology Co., Ltd. (Guangzhou, China). The DESeq package in R was used to normalize the gene expression profile and identify the differentially expressed genes (DEGs) among different groups. *p* > 0.05 and the absolute log 2 fold change (log 2 FC) between two groups larger than one were used as criteria for significantly differentially expressed genes. By using hierarchical clustering analysis, the miRNAs were aggregated into different colored regions representing different clustering information with the RPKM value of the differential miRNAs.

### 2.7. Quantitative Real-Time PCR

Total RNA was isolated from hippocampal tissues of CPP model animals or mouse hippocampal neuronal precursor HT22 cells. HT22 cells were classified into Con, Meth, Meth + Rhy-H9c2 coincubation (Rhy + Meth), and Meth + Con-H9c2 coincubation (Con + Meth) groups. H9c2 cells were incubated in a transwell chamber of six-well plates and coincubated with Meth-treated HT22 cells after administration of Rhy or PBS. The quality and integrity of the total RNA were evaluated by ND-1000 Nanodrop. A reverse transcription system (Accurate Biotechnology, Changsha, China) was used to convert messenger RNA to complementary DNA (cDNA) and RT-PCR performed with SYBR Green PCR master mix (Accurate Biotechnology, Changsha, China) using a LightCycler96 real-time PCR system (Roche, Basel, Switzerland). The threshold cycle (Ct) was defined as the fractional cycle number at which the fluorescence passed the fixed threshold. PCR was performed as follows: (1) 95°C for 30 s; (2) 95°C for 5 s, followed by 60°C for 30 s (repeat (2) for 40 cycles). U6 was used as an internal control to normalize expression of miRNA. Relative expression levels were calculated using the 2^−ΔΔCT^ method. Bulge-loop™ miRNA RT-PCR primer sets (one RT primer and a pair of qPCR primers for each set) specific for miRNAs and the U6 primer were designed by RiboBio Biotechnology Co., Ltd. (Guangzhou, China). Online databases, miRDB (http://mirdb.org/), miRWalk (http://mirwalk.umm.uni-heidelberg.de/), and TargetScan (http://www.targetscan.org/vert_71/), on miRNA target prediction and functional annotation were used to predicted the target of key miRNA for further study.

### 2.8. Transfection and Overexpression of miRNA

MiR-183-5p-mimic and NC-mimic were constructed by RiboBio Biotechnology Co., Ltd. (Guangzhou, China). H9c2 cells were transfected with the mimic constructs in a transwell chamber of six-well plates using Lipofectamine 6000 reagent (Beyotime Biotechnology, Shanghai, China) and coincubated with methamphetamine-treated HT22 cells after administration of Rhy. Cells were divided into Con, Meth, Meth + Rhy-H9c2 coincubation (Rhy + Meth), Meth + NC-mimic-Rhy-H9c2 coincubation (NC + Meth), and Meth + miR-183-5p-mimic-Rhy-H9c2 coincubation (mimic + Meth) groups.

### 2.9. Dual-Luciferase Reporter Assay

To verify the relationship between miR-183-5p and NRG1, we inserted the 3′-UTR-wild type (WT) and 3′UTR-mutant type (MUT) region of NRG1 into a vector and transfected the vector into 293T cells. Luciferase activity was measured 48 h after transfection with the Dual-Luciferase® Reporter Assay System (Promega, Fitchburg, WI, USA). The influence of miR‐183-5p on NRG1 was computed as relative luciferase activity (renilla luciferase/firefly luciferase).

### 2.10. Western Blotting Analysis

The proteins of EVs were measured using BCA protein assay (Beyotime Biotechnology, Shanghai, China). Protein samples were electrophoresed on 10% sodium dodecyl sulfate-polyacrylamide gels and transferred to a polyvinylidene fluoride membrane. The membrane was blocked with 5% skim milk at RT for 2 h. Membrane was incubated with EVs markers CD63 (Abcam, Cambridge, MA, USA) and tumor susceptibility gene 101 (TSG101, Abcam, Cambridge, MA, USA) overnight at 4°C and incubated with secondary antibodies (BOSTER, California, USA) at RT for 2 h.

The hippocampal tissues or HT22 cells were lysed with RIPA lysis (Solarbio Life Science, Beijing, China) to acquire proteins. Protein samples were electrophoresed on 8% sodium dodecyl sulfate-polyacrylamide gels, whose loading amount was 50 *μ*g, and transferred to a polyvinylidene fluoride membrane. The membrane was blocked with 5% skim milk at 25°C for 2 h. Membranes were incubated with NRG1 antibody (Proteintech Group, IL, USA) and ErbB4 antibody (Cell Signaling Technology, Danvers, MA, USA), overnight at 4°C, and incubated with secondary antibody (BOSTER, California, USA) for 2 h at 25°C.

Protein was detected using enhanced chemiluminescence reagents (Millipore, Billerica, MA, USA) and the expressions of protein between different groups were compared by using ImageJ 1.51.

### 2.11. Statistical Analysis

Statistical analysis was performed by using GraphPad Prism 7. The experimental results were expressed as mean ± standard deviation (means ± SD). The data of each group was analyzed by one-way ANOVA method. When the variance was uniform, LSD method was used to compare the two groups; when the variance was not uniform, the Dunnett-t method was used for comparison. The experimental results were considered to have significant statistical differences at *p* < 0.05.

## 3. Results and Discussion

### 3.1. Characterization of EVs

**Figure 1:**
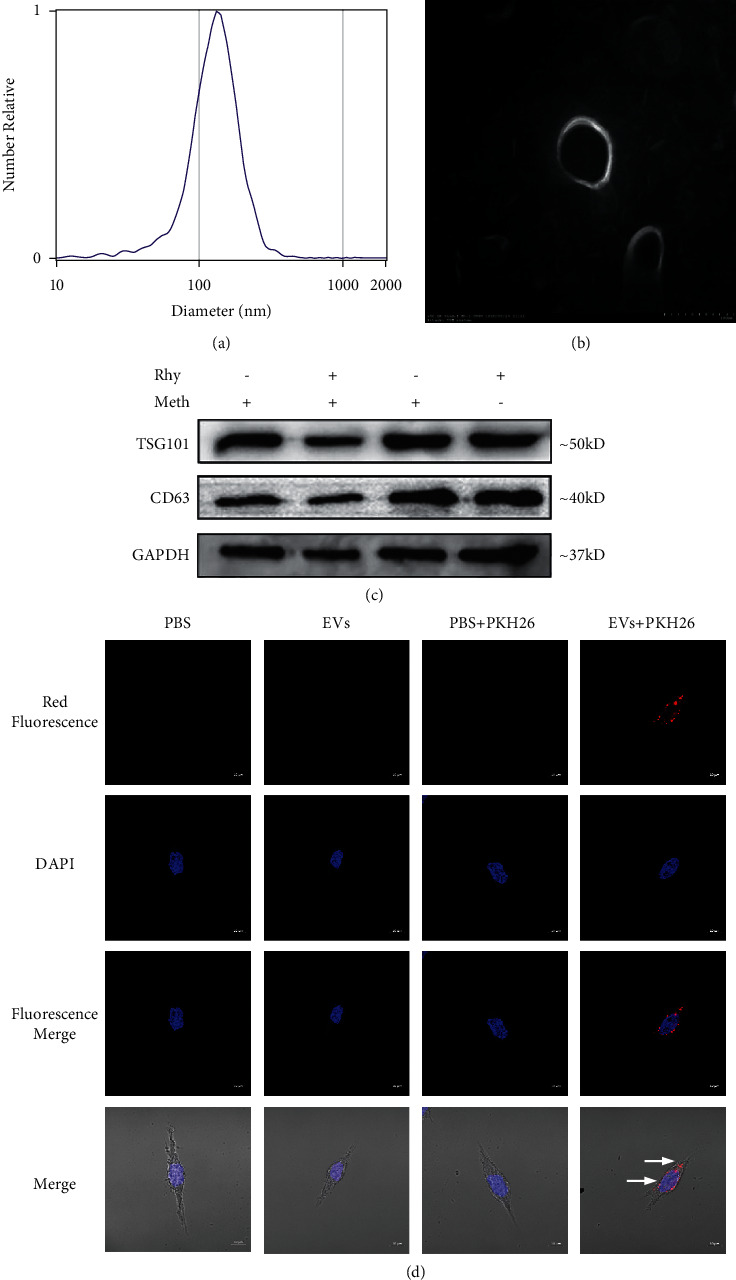
(a) Particle size distribution of EV nanoparticles. (b) Morphology of EVs observed using transmission electron microscopy (scale: 100 nm). (c) Western blot detection of TSG101 and CD63 proteins of EVs. Con: EVs extracted from cultured nontreated H9c2; Rhy + Meth: EVs extracted from cultured H9c2 treated with 250 *μ*M Meth for 48 h and 100 *μ*M Rhy for 24 h; Meth: EVs extracted from cultured H9c2 treated with 250 *μ*M Meth for 48 h; Rhy: EVs extracted from cultured H9c2 treated with 100 *μ*M Rhy for 24 h. (d) PKH26-labeled EVs secreted by H9c2 are taken up by neuronal HT22 cells (scale: 10 *μ*m).

EVs, secreted by H9c2 cells subjected to different treatments, with a diameter of ∼124.3 nm exhibited a typical ovoid-shaped bilayer membrane structure, as observed using NTA and TEM (Figures 1(a) and 1(b)). The treatment doses of Rhy and Meth were determined by MTT (Table S1). Furthermore, the surface marker proteins of EVs, TSG101 and CD63, were detected in EVs via western blot (Figure 1(c)). These results show these small molecules are EVs, which could be used for further study. To determine whether H9c2-EVs can be taken up by the neuronal precursor cells HT22, we used in vitro coincubation. After 12 h of coincubation, the presence of PKH26-labeled EVs (red) in the cytoplasm of HT22 was observed in confocal images, indicative of cardiomyocytes-EVs uptake by neurons (Figure 1(d)).

### 3.2. Rhy-EVs Inhibited Meth-Induced CPP Effect in Mice

**Figure 2:**
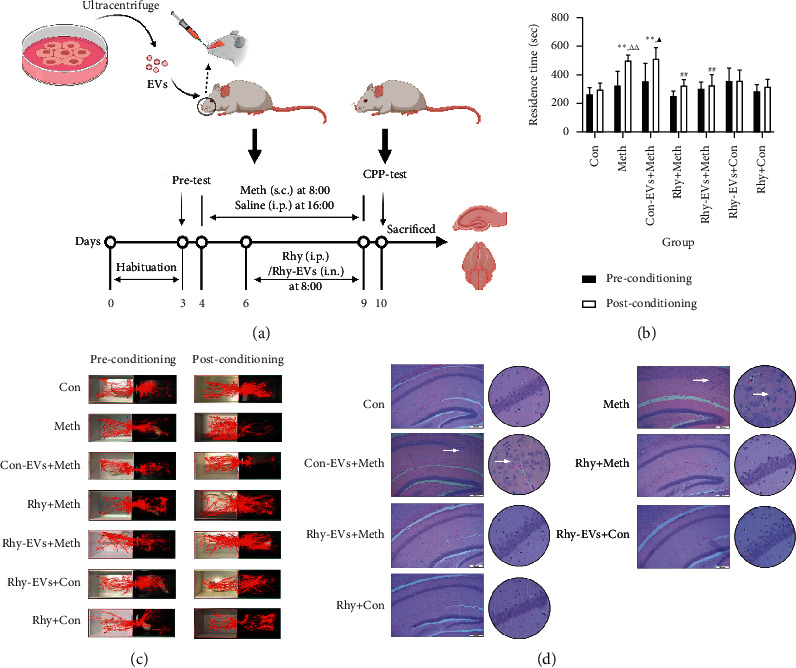
(a) Schematic protocol of CPP testing (created with BioRender.com). (b) Time spent in the drug compartment during preconditioning and postconditioning (*n* = 6, *t*-test for preconditioning vs. postconditioning, one-way ANOVA for comparison between groups on preconditioning and postconditioning). Data are presented as means ± SD, ^*∗∗*^*p* < 0.01 vs. Con group, ^##^*p* < 0.01 vs. Meth group, ^△△^*p* < 0.01 vs. preconditioning Meth group, and ^▴^*p* < 0.05 vs. preconditioning Con-EVs + Meth group. (c) Representative head trajectories of mice in the CPP apparatus in pre- and postconditioning phases. (d) Histopathological analysis of hippocampal tissues via hematoxylin-eosin (H&E) staining (scale: 200 *μ*m).

Based on evidence that Rhy plays an inhibitory effect in Meth dependence, next we tested Rhy-EVs effects on CPP-induced by Meth. Schematic protocol of CPP test is shown in Figure 2(a). Residence time, locomotor activity (recorded by head trajectories), and H&E staining of hippocampal tissues were examined for evaluating drug dependence in the CPP model. The Panlab Smart 3.0 software tracked the mice heads and recorded head trajectories. A total recording time of 15 min for each mouse was divided into three 5-minute segments. The total recording time was used to examine residence time, and the second segment was chosen to examine locomotor activity. As shown in Figures 2(b) and 2(c), we observed no significant differences in time spent and head trajectories in the drug compartment among all groups before intervention (*p* > 0.05). After Meth administration and training for 6 consecutive days, mice in the Meth group spent significantly more time in the drug compartment than those in the Con group (*p* < 0.01). The inhibitory effect of Rhy and EVs secreted by Rhy-treated H9c2 cells on CPP were evident in the Rhy + Meth and Rhy-EVs + Meth groups (*p* < 0.01). In contrast, EVs secreted by PBS-treated H9c2 cells (Con-EVs + Meth group) exerted no significant effect (*p* < 0.05 vs. Con group). Moreover, the control model treated with Rhy (Rhy + Con group) and EVs secreted by Rhy-treated H9c2 cells (Rhy-EVs + Con group) have no effect on CPP measures. We found normal hippocampal CA1 cells were round and oval, while those in the Meth group were necrotic and arranged loosely with smaller nucleoli. The damage to the hippocampus of the Con-EVs + Meth group was slightly less than that of the Meth group. The Rhy-EVs + Meth treatment group displayed an obvious decrease in neuronal degeneration in hippocampal CA1 compared to the Meth group (Figure 2(d)). Our data strongly indicate that Rhy-EVs effectively reverse the CPP effect and neuronal damage in hippocampal CA1 of mice induced by Meth dependence.

### 3.3. Screening the Differentially Expressed miRNAs in H9c2-EVs and Demonstrating the Expression of miRNA via qPCR In Vivo and In Vitro

**Figure 3:**
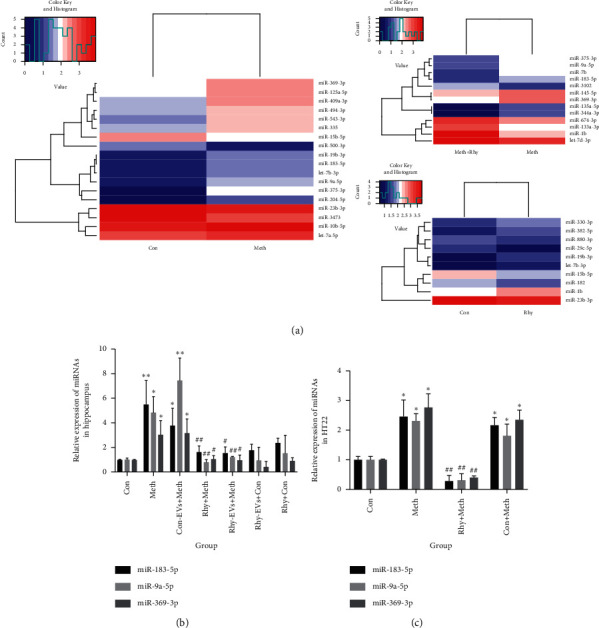
(a) Heatmap analysis of differentially expressed miRNAs in H9c2-derived EVs from different treatment groups. Blue represents low expression and red represents high expression. (b) Fold changes in miR-183-5p expression in hippocampal tissues (*n* = 3, one-way ANOVA, *p* < 0.05). Data are presented as means ± SD, ^*∗*^*p* < 0.05, ^*∗∗*^*p* < 0.01 vs. Con group; ^##^*p* < 0.01 vs. Meth group. (c) Fold changes in miR-183-5p expression in HT22 cells (*n* = 3, one-way ANOVA, *p* < 0.05). Data are presented as means ± SD, ^*∗∗*^*p* < 0.01 vs. Con group; ^##^*p* < 0.01 vs. Meth group. Rhy + Meth: Meth + Rhy-H9c2 coincubation group; Con + Meth: Meth + Con-H9c2 coincubation group.

Next, we used the small RNA sequencing test to examine the role of miRNA in EVs. As shown in Figure 3(a), the heatmap data displayed that 4 miRNAs (miR-375-3p, miR-369-3p, miR-9a-5p, and miR-183-5p) were significantly differentially expressed in the absence of the effect of Rhy, showing upregulation in the Meth group and downregulation in the Rhy + Meth group. Previous experiments in this study have confirmed that EVs transferred its contents to recipient cells and EVs can be taken up by recipient cells. Therefore, it is reasonable to hypothesize that the changes of miRNA in target cells or organs are consistent with those in EVs. Here, we used qPCR to validate the differentially expressed miRNAs of EVs both in vivo and in vitro. In accordance with small RNA sequencing results, qPCR revealed upregulation of miR-183-5p, miR-9a-5p, and miR-369-3p after Meth administration and downregulated after Rhy intervention both in vivo and in vitro (Figures 3(b) and 3(c)). However, the expression of miR-375-3p did not show any statistical difference (Figure S1A). Take three miRNAs into consideration, the expression of miR-183-5p in hippocampus was the most significantly elevated in Meth group, and its expression trends were consistent with the results of the previous behavioral test. Therefore, we selected miR-183-5p for further experiments.

The qPCR results verified that the level of miR-183-5p was reduced in the Con-EVs + Meth group compared to the Meth group, but it was significantly different from the Con group (*p* < 0.05). It indicates that Con-EVs has an insignificant therapeutic effect on Meth dependence and does not cause a substantial change in the expression of miR-183-5p (*p* > 0.05 vs. Meth group). However, compared to Meth group, the expression of miR-183-5p in Rhy-EVs + Meth group was significantly decreased (*p* < 0.01), identifying that Rhy-EVs has a significant effect on resisting Meth dependence. These findings confirm the hypothesis that Rhy exerts a neuroprotective effect by changing the expression of miR-183-5p in EVs.

### 3.4. Target Gene and Pathway Analysis of miR-183-5p

Hence, we continued to used miRDB, miRWalk, and TargetScan to identify miR-183-5p target genes related to drug addiction and those target genes of miR-183-5p enriched for brain-related pathways were selected for further study. Based on predictions from these online databases, miR-183-5p expressed in the brain and its target gene, NRG1, an epidermal growth factor (EGF)-like protein implicated in neural development and brain activity homeostasis, were highlighted as likely candidates. TargetScan indicated that NRG1 mRNA contained eight matched nucleotides with miR-183-5p at position 1399-1406 in the 3′UTR (Figure S1B).

### 3.5. H9C2-EVs Protect against Meth-Induced Dependence in Mouse Brain and HT22 Cells through Upregulating NRG1

**Figure 4:**
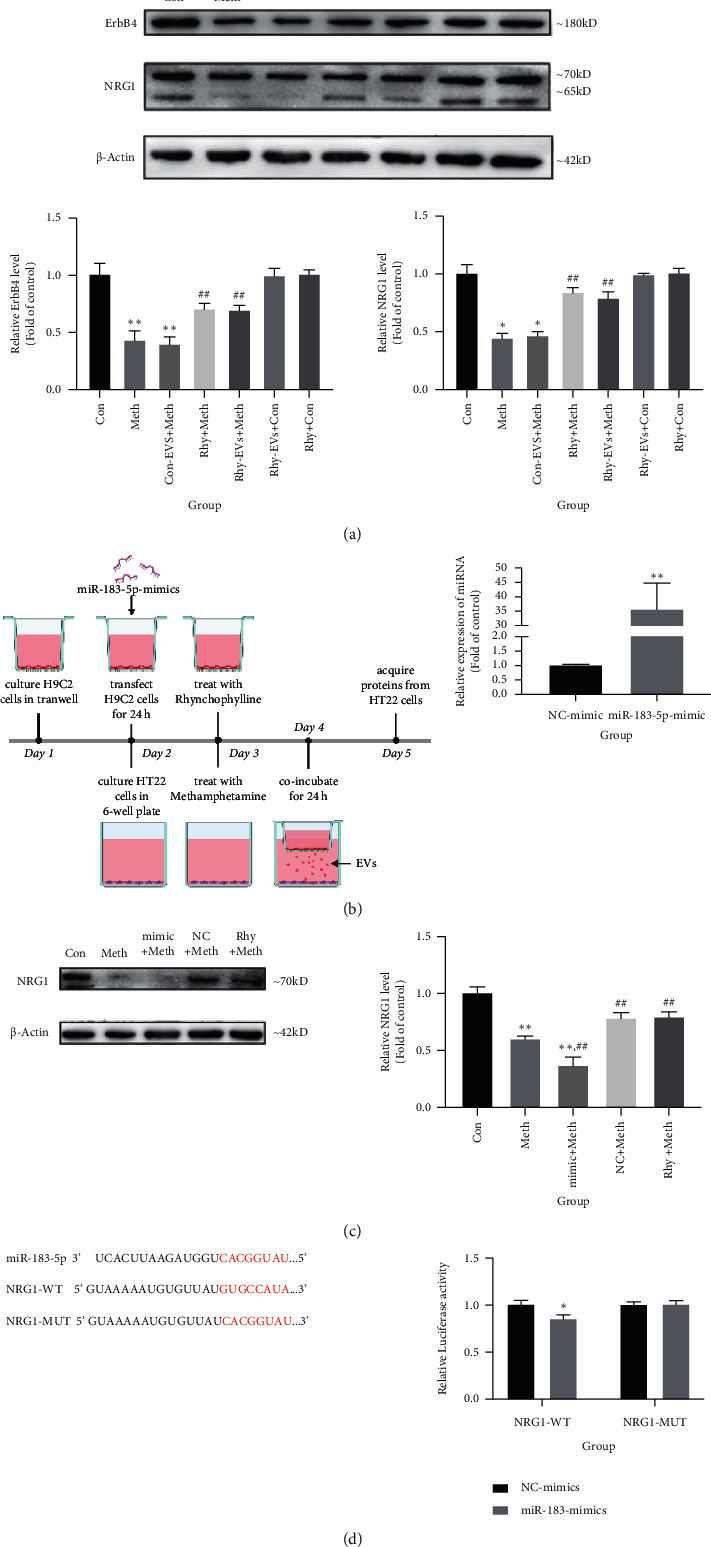
(a) Western blot detection of relative expression of NRG1 and ErbB4 in hippocampal tissues (*n* = 4, one-way ANOVA, *p* < 0.05). Data are presented as means ± SD, ^*∗∗*^*p* < 0.01 vs. Con group; ^##^*p* < 0.01 vs. Meth group. (b) Schematic protocol of transfection (created with BioRender.com). H9c2 cell transfection efficiency of overexpressed miR-183-5p (miR-183-5p-mimic). Data are presented as means ± SD, ^*∗∗*^*p* < 0.01 vs. NC-mimic group. (c) Relative expression of NRG1 in HT22 cells detected via western blot (*n* = 4, one-way ANOVA, *p* < 0.05). Data are presented as means ± SD, ^*∗∗*^*p* < 0.01 vs. Control, ^##^*p* < 0.01 vs. Meth group. Mimic + Meth: H9c2-transfected miR-183-5p-mimic with Rhy + Meth coincubation; NC + Meth: H9c2-transfected miR-NC-mimic with Rhy + Meth coincubation; Rhy + Meth: H9c2 with Rhy + Meth coincubation group. (d) Dual-luciferase assay of miR-183-5p targeting effects on NRG1 3′-UTR (*n* = 3, *t*-test, *p* < 0.05). Data are presented as means ± SD, ^*∗*^*p* < 0.05.

We conducted a series of experiments to further examine the relationship between EVs-containing miR-183-5p and NRG1. Western blot analysis revealed elevated NRG1 expression in hippocampal tissues of CPP model animals. After administration of Meth, NRG1 protein levels in hippocampal tissues were significantly decreased compared to the Con group, whereas significantly increased NRG1 proteins were observed with Rhy and Rhy-EVs treatments (Figure 4(a)). These findings show that miR-183-5p of Rhy-EVs may inhibit the Meth addiction through modulating NRG1.

In order to confirm the role between miR-183-5p and NRG1 in the inhibitory effect of Rhy on Meth addiction, we further used miR-183-5p-mimic to treat H9c2 cells. Firstly, to determine cellular expression patterns, H9c2 cells were transfected with miR-183-5p-mimic and then coculture with HT22 cells. The transfection efficiency of the mimic in H9c2 cells was verified using qPCR (Figure 4(b)). Administration of Meth led to a significant decrease in NRG1 expression in HT22 cells, but the effect was completely reversed in coincubation with Rhy-EVs. Next, we coincubate the EVs, separated from H9c2 cells treating with miR-183-5p-mimic and Rhy, with HT22 cells. The result displayed further downregulation of NRG1 in HT22 cells compared to the Meth treatment group, suggesting that H9c2-EVs regulate NRG1 expression through miR-183-5p (Figure 4(c)). These results also demonstrated the regulation of miR-183-5p-NRG1 in in vitro meth addiction model. In addition, a dual-luciferase reporter system also demonstrated that NRG1 is a direct target of miR-183-5p. The relative luciferase activity decreased when miR-183-5p-mimics were cotransfected with NRG1-WT, but not with the NRG1-MUT, indicating that NRG1 is a direct target of miR-183-5p (Figure 4(d)). Together, we concluded that miR-183-5p negatively modulated NRG1 by directly targeting its 3′-UTR and Rhy-EVs inhibited the Meth dependence via miR-183-5p/NRG1 pathway.

## 4. Conclusions

Previous researches have generated tremendous interest in the connection of CNS and heart in drug addiction [4, 26, 27]. However, the underlying crosstalk and mechanisms between these two systems have not been fully elucidated. Recently, the EVs are served as an effective communication by transferring its contents, including miRNAs, to neighboring or specific cells between the CNS and heart [28]. In this study, we found that Rhy-EVs can inhibit the CPP effect induced by Meth dependence, as well as Rhy treatment. Moreover, we further identified that miR-183-5p mediated the inhibition of NRG1 expression and that the transfer of exosomal miR-183-5p-mimic, produced by H9c2 cells, to HT22 cells exerted inhibitory effect of NRG1 expression. These results indicate that Rhy-EVs inhibited the Meth dependence via miR-183-5p/NRG1 pathway and provide evidence of EVs as the communicated mediators for the antidrug addictive effect of Rhy treatment.

H9c2 cell line is isolated from rat embryonic heart, which has similar morphological structure and electrophysiological characteristics to primary cardiomyocytes. Relative studies have shown that the biological properties of EVs secreted by H9c2 cells and primary cardiomyocytes are similar to a certain extent [29]. The small molecules collected from supernatant fractions of H9c2 cells were identified as EVs and our results indicated that Rhy-treated H9c2 EVs significantly decreased the time spending in the drug compartment, as well as Rhy administration. And H&E staining results also confirmed that Rhy-treated H9c2-EVs exerted an apparent therapeutic effect against Meth-induced damage in mouse brain, while PBS-treated H9c2-EVs group showed a subtle degree of protective effect.

Given the data, both Rhy and Rhy-treated H9c2 EVs played an inhibitory effect on drug addiction, and it has recently been shown that EVs derived from peripheral cells can cross through the blood-brain barrier and be taken up by target cells, which could ameliorate neurodegeneration [30]. Consistent with the literature, we found that EVs isolated from supernatant fractions of H9c2 cells were successfully taken up by neuronal HT22 cells. Therefore, these data offer the evidence that EVs can deliver the contents of Rhy treatment into brain and suggest that EVs act as mediators during the process of drug addiction and efficient carriers from heart to brain after Rhy intervention.

Particularly, EVs and EVs-containing miRNAs are involved in the regulation of multiple physiological and pathological activities, such as regulating neuronal development and regeneration, participating in cell-cell communication in neurodegenerative diseases, modulating synaptic functions and synaptic functions in the CNS [17]. A previous study has reported that 169 plasma EVs miRNAs levels were changed in humans actively using Meth, and three miRNAs of the humans actively using Meth significantly related with clinical features of Meth use and target prediction with these miRNAs demonstrated pathways implicated Meth use, including cardiovascular disease and neuroinflammation [31]. Instinctively, EVs could serve as a potential source of delivering behavior-altering miRNAs from peripheral systems to critical brain regions controlling Meth dependence and therefore require a closer examination.

To investigate the protective mechanisms of action of cardiac-derived EVs miRNAs under conditions of Rhy intervention, we analyzed the miRNA profile in vivo and then predicted and examined the candidate target genes and potential signaling pathways of differentially expressed miRNAs. Our current small RNA sequencing analysis demonstrated that four miRNAs displayed significant different expression in the Con group, the Meth group, and the Meth + Rhy group. However, Meth is a controlled substance, and it is difficult to obtain enough total RNA of isolated EVs. Since the miRNAs of the EVs could be absorbed by the recipient cells, qPCR was used to detect and verify the expression of differential miRNAs in hippocampus and HT22 cells. We observed that the expression trends of miR-183-5p, miR-9a-5p, and miR-369-3p via qPCR were consistent with the results of small RNA sequencing test. In addition, miR-183-5p was the most significantly enhanced compared to the other two miRNAs, and miR-183 is also primarily involved in cell communication, protein kinase activity regulation, and adrenergic signal in cardiovascular system [32]. Meanwhile, miR-183-5p can reduce ischemic injury in CNS [33], increase neurite outgrowth, and mediate neuroprotection of DA neurons in vitro and in vivo [34]. These findings indicate that miR-183-5p is validated as a potential key miRNA for neurological diseases; therefore we further explored the mechanisms of EVs-containing miR-183-5p during the Meth addiction and Rhy treatment and it is the first study to support the role of miR-183-5p in drug addiction.

Based on predicted data from miRDB, miRWalk, and TargetScan, we focused on the gene NRG1, one of the downstream pathways of miR-183-5p. NRG1 is not only an EGF-like protein implicated in neural development and brain activity homeostasis [35] but also an important factor for memory formation and an indicator of Meth dependence [36], which regulates nerve cell differentiation, neuron migration, neurotransmission, neurite outgrowth, and synaptic activity and protects neurons under various pathological conditions [37]. Furthermore, recent evidence also suggested that NRG1 is an appropriate candidate gene for Meth-induced psychosis and overexpression of NRG1 type III modulated the behavioral tests induced by Meth [38, 39]. In particular, neuregulins (NRGs) are cell-cell signaling proteins that act as ligands for receptor tyrosine kinases of the ErbB family. In the hippocampal CA1 region, ErbB4 expressed in GABAergic neurons plays essential roles in the central and peripheral nervous systems [35, 40]. Further studies revealed that NRG1-ErbB4 signaling pathway regulates expression of NMDA, GABA, and ACh receptors, which are genetically linked to psychiatric disorders [37]. Additionally, this signaling pathway could modulate anxiety-like behavior and GABA release [41], prevent neuronal cell death during functional recovery after traumatic brain injuries (TBI) [42], and also improve performance in spatial learning and memory, attenuating hippocampal CA1 neuronal loss and apoptosis [43]. It is well studied that both GABAergic systems and synaptic plasticity are necessary for the process of Meth addiction formation [44], suggesting that the NRG1-ErbB4 signaling pathway may be related to the underlying mechanism of drug addiction. However, the association of NRG1-ErbB4 signaling with Meth addiction has not been established to date.

According to these observations, we examined whether NRG1 is a target of miR-183-5p. Compared to the Meth group, neuronal miR-183-5p was downregulated after administration of Rhy-EVs both in vivo and in vitro. In contrast, upregulation of hippocampal NRG1 and ErbB4 levels was found in Meth + Rhy-EVs group via western blot analysis, supporting the proposal that Rhy-EVs protect against damage caused by Meth via targeting NRG1-ErbB4 signaling pathway. As Meth is the controlled and illicit drug, in the present study, we identified whether Rhy inhibited Meth-induced CPP through miR-183-5p/NRG1/ErbB4 signaling pathway in vitro, which can reduce the Meth usage to obtain the data. The findings indicated that, for coculture H9c2 cells, transfected with miR-183-5p-mimic and Rhy, with HT22 cells, miR-183-5p could be elevated and NRG1 further decreased in HT22 cells. Consistent with these results, the data from the dual-luciferase reporter assay verified that the NRG1 3′-UTR is a direct inhibitory target of miR-183-5p.

The present study has two limitations. One concern is that the regulation of miR-183-5p/NRG1 was only confirmed in vitro. Inevitably, in order to further confirm the role of miR-183-5p/NRG1/ErbB4 pathway in the process of drug addiction, it is necessary to detect and verify the expression of miR-183-5p/NRG1/ErbB4 in vivo through brain stereotaxic. Another limitation is that in this study we just focused on the hippocampus without studying other brain areas related to learning and memory. Future studies of our group will launch to identify the role of miR-183-5p/NRG1/ErbB4 pathway and the effect of Rhy-EVs on other brain regions in drug addiction.

Our collective findings demonstrated for the first study that Rhy-EVs exert neuroprotective effects on the hippocampus in Meth-dependent mice. Results suggest that Rhy delivered via EVs to Meth-dependent brain plays a therapeutic role partly via regulation of EVs-encapsulated miR-183-5p/NRG1 signaling pathway. Also, the research presented here clearly demonstrates the promising therapeutic potential of exosomes derived from H9c2-treated Rhy in the treatment of drug addiction. Finally, these findings will help guide further studies to other peripheral systems, for instance, liver and kidney, for development of TCM used for treating drug addiction.

## Data Availability

The small RNA sequencing data used to support the findings of this study have been deposited in the Sequence Read Archive Repository (PRJNA673056).

## Supplementary Materials

Supplementary Materials Table S1: detailed information of relative cell viability. Figure S1: the expression of miR-375-3p and predicted consequential pairing of NRG1 and miR-183-5p.
