# Supplementary material for: Extracellular Vesicle-Encapsulated miR-183-5p from Rhynchophylline-Treated H9c2 Cells Protect against Methamphetamine-Induced Dependence in Mouse Brain by Targeting NRG1
Source: Evid Based Complement Alternat Med. 2021 Aug 26;2021:2136076. doi: 10.1155/2021/2136076 (PMC8416368; doi:10.1155/2021/2136076)
Supplement: Supplementary Materials — Table S1: detailed information of relative cell viability. Figure S1: the expression of miR-375-3p and predicted consequential pairing of NRG1 and miR-183-5p. [file 2136076.f1.docx]

**Supporting Information**

**Table S1**

**Table S1.** The effect of Rhy and Meth on the relative cell viability of H9c2 (n = 3).

| Consentration  (mmol/L) | Relative cell viability in Rhy (%) | Relative cell viability in Meth (%) |
| --- | --- | --- |
| 0  0.05  0.10  0.15  0.20  0.25 | 100±0.0  95.52±0.22  94.09±1.10  88.23±1.28  84.56±1.36  74.68±1.81 | 100±0.0  95.79±0.57  97.18±1.09  94.68±1.58  98.64±2.06  105.14±2.06 |

**Figure S1**


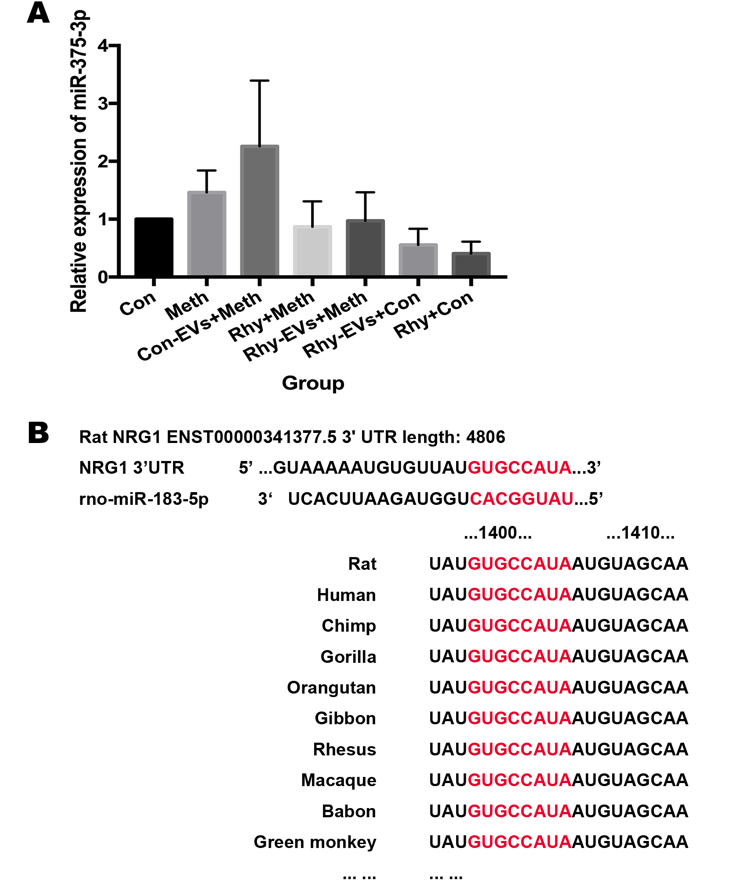


Figure S1. (A) Fold changes in miR-375-3p expression in hippocampal tissues (n=3). Data are presented as means ± SD, no significant vs. Con group or Meth group. (B) Predicted consequential pairing of NRG1 and miR-183-5p.
